# Nurses and the acceptance of innovations in technology-intensive contexts: the need for tailored management strategies

**DOI:** 10.1186/s12913-021-06628-5

**Published:** 2021-07-03

**Authors:** Chiara Barchielli, Cristina Marullo, Manila Bonciani, Milena Vainieri

**Affiliations:** Institute of Management and EMBEDS Department Sant’Anna School of Advanced Studies, Pisa, Italy

**Keywords:** Nurses, Innovation, Technology acceptance, Technology-intensive contexts

## Abstract

**Background:**

Several technological innovations have been introduced in healthcare over the years, and their implementation proved crucial in addressing challenges of modern health. Healthcare workers have frequently been called upon to become familiar with technological innovations that pervade every aspect of their profession, changing their working schedule, habits, and daily actions.

**Purpose:**

An in-depth analysis of the paths towards the acceptance and use of technology may facilitate the crafting and adoption of specific personnel policies taking into consideration definite levers, which appear to be different in relation to the age of nurses.

**Approach:**

The strength of this study is the application of UTAUT model to analyse the acceptance of innovations by nurses in technology-intensive healthcare contexts. Multidimensional Item Response Theory is applied to identify the main dimensions characterizing the UTAUT model. Paths are tested through two stage regression models and validated using a SEM covariance analysis.

**Results:**

The age is a moderator for the social influence: social influence, or peer opinion, matters more for young nurse.

**Conclusion:**

The use of MIRT to identify the most important items for each construct of UTAUT model and an in-depth path analysis helps to identify which factors should be considered a leverage to foster nurses’ acceptance and intention to use new technologies (o technology-intensive devices).

**Practical implications:**

Young nurses may benefit from the structuring of shifts with the most passionate colleagues (thus exploiting the social influence), the participation in ad hoc training courses (thus exploiting the facilitating conditions), while other nurses could benefit from policies that rely on the stressing of the perception of their expectations or the downsizing of their expectancy of the effort in using new technologies.

**Supplementary Information:**

The online version contains supplementary material available at 10.1186/s12913-021-06628-5.

## Introduction

Technological innovations are an opportunity to address the challenges of modern health, but only if accompanied by a transformation in behaviour and habits of people [1].

Indeed, innovations refer to “the design, the early adoption and implementation of new services, ideas, or modes of action [ …] that are significant in order to improve or reform them” [2].

Many technological innovations have been introduced in healthcare over the years, and healthcare workers have frequently been called upon to become familiar with technological innovations that pervade every aspect of their profession, changing their working schedule, habits, and daily actions. This appears to be even more important in turbulent contexts, such as the current pandemic, where all these factors have undergone a rapid acceleration [3].

For nurses, technological competence and nursing care are two seemingly distant elements that are “harmoniously coexisting”, according to Locsin’s theory [4]. In fact, nursing is based on the knowledge of the “patient as a whole” (Ibidem) resulting from continuous observation and technology supports the knowledge process both in depth and in promptness [5]. Technological innovations therefore allow nurses to be closer to people since allow a deeper knowledge of their patients.

When nurses deal with technological innovation and purposively adopt it, they can be considered successful innovators as they focus on the opportunity the technological innovation gives them, in order to minimise the risks [6] for themselves and for the patients they take care of.

In an analysis about innovations led by nurses, Hughes [7] emphasizes that these “innovators” share well-defined characteristics, such as a high level of tenacity and determination, motivation to learn, and a strong desire to earn qualifications which confer social recognition. Kouta [8] adds to this description that nurses’ propensity to adopt technological innovations is also supported by the desire to provide a more effective care for their patients.

Technology-intensive healthcare contexts, such as robotic surgery units, intensive care units or remote-control units for telemetries, are characterized by the continuous introduction of new technologies. Items, machinery and equipment are constantly renewed, especially in some areas and for some types of interventions and require contextual knowledge and tailored management strategies to maximise their efficiency. In these contexts, knowledge of how to use the new equipment and the ability to convert such knowledge into practical care are key aspects for management to evaluate. Nurses represent the most numerous components of human resources in healthcare organizations and are involved in every stage of the care process, from prevention to the management of chronic conditions. Investigating the determinants of acceptance of innovations by nurses operating in technology intensive contexts, such as, e.g. ICU, operating theatres and biotelemetry stations, appears particularly relevant when, in situations of quick (and unexpected) change, an increase in organizational flexibility is needed.

It is important that healthcare managers understand the determinants of acceptance nurses have toward new technologies in these specific settings, and, in particular, the differences occurring between older and younger workers. Generational characteristics and differences among the staff can be enriching, but must be leaded [9]. Generational differences in acceptance levels and determinants between age groups illustrate why management strategies need to be tailored to different types of employees, or to achieve the highest possible degree of acceptance towards the innovations that are being introduced.

Drawing on the Unified Theory Of Acceptance And Use Of Technology (UTAUT), the objective of this study is to investigate the determinants of technology acceptance by nurses operating in technology-intensive healthcare contexts and to test the effect of professionals’ seniority on the model.

### Theoretical background

Several theoretical models have been developed to explain the determinants of acceptance of a new technology by users. More specifically, authors tried to explain technology acceptance as a decision to actually use an innovation, and to understand which factors justify and encourage its effective use.

The knowledge of these mechanisms and factors is in fact strategic to support both the development and the implementation of a technology within an organization.

Among these models, the Unified Theory of Acceptance and Use of Technology (Unified Theory of Acceptance and Use - UTAUT), developed by Venkatesh and Colleagues [10], is considered to be the most sensitive and up to date.

The UTAUT model refers to and integrates 8 theories and models and provides a framework to describe and interpret the dynamics of the acceptance of technology (Table 1).

**Table 1:** Theories and models integrated in the UTAUT framework

Reference	Theoretical model	Main concept (s)
Fishbein and Ajzen (1975) [11]	The *theory of reasoned action* – TRA	Behaviours mediated by individuals’ predisposition towards specific actions
Ajzen (1991) [12]	The *theory of planned behaviour* -TPB	Perceived behavioural control
Davis (1985) [13] Venkatesh and Davis, 1996) [14]	The *technology acceptance model* – TAM	Perceived usefulness Perceived ease of use
Taylor and Todd (1995a; 1995b) [15, 16]	The model that combines TAM and TBP	TBP better explains behavioural intention
[17]	The *motivational model* -– MM	Use of innovations as based on extrinsic and intrinsic motivations
[18]	The *model of PC utilization* - MPCU	Focus on current behaviour
Bandura (1986) [19]	The *social cognitive theory* - SCT	Internal personal factors Behavioural factors Environmental events
Rogers (1962) [20]	The *diffusion of innovation theory* - DOI	Innovation adoption as a process

In 1975 Fishbein e Ajzen [11], via their TRA, point out that behaviours are mediated by the predisposition that the individual has towards that specific action. They define behaviour as the result of the intention to assume a specific conduct (*behavioural intention*). This latter intention is determined by both personal attitude and subjective norms. The personal attitude towards behaviour is the attitude an individual has in adopting or not adopting a specific behaviour, that is from the judgment (positive or negative) that a person attributes to a particular behaviour based on their own beliefs and personal assessments. The subjective norms represent the influence that the opinions of others exercise on the choices of the individual, and that it depends on the convictions that the reference people of the individual have and the willingness that the individual has in adapting their behaviour to the expectations of their reference people.

The theory of planned behaviour by Ajzen [12] represents an extension of the previous theory of reasoned action, as it introduces a new variable relating to perceived behavioural control. This last variable, which affects the intention and actual behaviour of a particular behaviour, is based on an individual’s perception of being able to implement the desired behaviour and is determined by the availability of resources, by the opportunities and skills available and by the meaning that the individual attaches to those resources, opportunities and skills to achieve the desired results.

Davis’ [13] technology acceptance model is designed to explain the general determinants of computer acceptance and is currently one of the most widely used models in innovation acceptance studies. This theory is based on two main constructs: the *perceived usefulness* of the technology defined as the idea that the user has with respect to the probability that the use of the technology is useful or not for himself, and the *perceived ease of the use* of the technology which corresponds to what the user expects to be able to use it effortlessly. Both perceived utility and ease of use influence the intention to use the specific technology.

These studies have refined over time, including increasingly larger points of view, following the unstoppable phenomenon of the expansion of the use of technology in every area of life. What they really lack is time perspective: they focus on the reality they describe, not taking into account the exponential growth in the number of technologies and their pervasiveness in societies, as we know it nowadays.

The motivational model (MM) explicit that the use of an innovation is based on extrinsic and intrinsic motivation. The first is defined as the perception that the use of innovation is instrumental to achieve better results, while the second is defined as the perception that the use of innovation is in itself a value, without any further purpose. The perceived usefulness of technology is therefore an extrinsic motivation for its use, while the perceived pleasure of technology itself (*perceived enjoyment*) is an intrinsic motivation.

The model of PC utilization (MPCU) focuses on current behaviour, excluding the intention to use it, as well as the habit to do so because it is directly connected with actual use, and stresses how the characteristics of the work, social factors, the long-term consequences and complexity influence computer use more.

The social cognitive theory explains human action through the interaction of three factors: internal personal factors (cognitive, affective and biological), behavioural factors (based on use, performance and behavioural adoption in a specific context) and environmental events (physical and social). These three causal factors are considered to influence each other in a bidirectional way. In the application of cognitive social theory to interpret the use of technology are also used self-efficacy constructs, expected performance results, anxiety and expectations of personal result.

The diffusion of innovation theory, DOI, explains that the process of adopting an innovation goes through several stages ranging from knowledge of the innovation itself, to the persuasion of its potential usefulness, to the decision whether to adopt it or reject it, its implementation and the confirmation of the use of innovation. The author of this theory, Rogers, highlights that usually in this process there is a minority of people who adopt it immediately (innovators and early adopters), the majority of people who adopt it more or less promptly (early majority and late majority), and a residual part of latecomers (laggards).

In the interest of being comprehensive, there are two works on UTAUT that have to be mentioned, namely the works of Williams et al. [21] and the work of Dwivedi et al. [22], a meta-analysis on meta-UTAUT.

The first work, through a systematic literature review, proves that UTAUT has been used to examine general purpose systems and specialized system, proving itself as an umbrella-framework under which it is possible to study and deepen the knowledge of a variety of realities, among which healthcare also finds its place. The authors indicate that the users’ acceptance of technology is an evergreen management challenge, enumerating the countless citations and references to the UTAUT theory but not giving practical indication on the information that it can provide in terms of management policies’ crafting. The second work pushes knowledge towards a modified theory based on 162 previous literature works [22]. Meta-UTAUT theorizes that attitudes towards the acceptance of technology are to be considered as part of the characteristics that every individual has. It is important to mention that the individual sphere is here prominent in respect of the previous theories. The authors state that this new theory is far from to be a theoretical alternative to the classic UTAUT when it comes to investigate the adoption of emerging, new technologies.

However, most of these studies did not put emphasis on the significance that each construct has to be contextualized in the nursing reality. Past studies illustrated the dynamics of technology acceptance in the nursing context, but they revolved around the acceptance and use of the Electronic Patient Records (EPRs) [23–25]. In addition to these studies which are explanatory to a circumscribed aspect, there are others which focus on the acceptance of a very specific type of technology: (i) the acceptance of Use Telemedicine Technology (eICU) and the predictors of nurses’ use of [26] that investigate how the perceived usefulness of its use in relation to the years worked in the hospital, (ii) the nurses’ Acceptance of Radiofrequency Identification Technology, where Norten [27] predicts the adherence to the protocols surrounding this new technology. The interest expressed in this work for the factors leading to the acceptance of a new technology is not based on prediction but on the detection of objective personnel characteristics and focuses in larger contexts, such as those mentioned above.

#### Nurses and the acceptance of innovations in technology-intensive contexts: hypotheses developmen

To investigate the mechanism of acceptance of technological innovations by nurses operating in technology-intensive healthcare contexts, the Unified Theory of Acceptance and Use of Technology (UTAUT) is applied [10]. This choice was motivated by the consideration that the UTAUT theoretical model includes in itself major theories and has been exhaustively tested on different technologies, including health technologies (e.g. EPRs, robotic assisted systems, telemedicine systems). In this study, the UTAUT framework is applied to explore which factors influence nurses’ intentions to use and their effective use of new technologies, and which of these factors can be used as leverage to amplify this latter. Healthcare organization is complex. So complex that it has been referred to as the “system of systems” [28]. The major component, nurses, are a pivotal asset to be known and understood, also with regards to the dynamics that occur in technology adoption. This knowledge will be of an extreme help for management, which will be able to leverage on the most important point in order to make the nursing part of the organizations prone to the acceptance of new technologies.

The main goal of this study is to provide insights on the mechanisms underlying the acceptance and the intention to use new technologies from nurses operating in technology-intensive contexts. It is known that equipment changes fast even inside the inpatient wards, but the most fast changing and technological intensive fringes like ICU, robotic surgery, remote telemetrics, are some of the fastest. It is not infrequent that a ventilator model changes within a year as not only producers are perfecting the existing equipment, but also because healthcare is close to the frontier of scientific discovery and always strives to go further, to the benefit of the health and longevity of the population. This aspect, typically characterizing technology intensive healthcare context becomes more important and widespread.

To the best of our knowledge, the UTAUT model has never been used for nursing in this specific technology-intensive healthcare context.

In this context, all the main constructs of the UTAUT model assume relevance and a peculiar meaning:

1. Performance Expectancy (PE) about a technology represents how much an individual believes that a given technology will help him to obtain advantages in the performance of his work. Nurses will deal with technology and accept it when they focus on the new opportunity they have to safeguard themselves and their patients [6].
2. Effort Expectancy (EE) represents how much an individual believes that the use of technology is easy. Past studies on technology adoption in the context of nursing, before using a technology nurses need to perceive that they can effectively use it [29], nurses who feel up to work with a new technology are more likely to use it [30].
3. Social Influence (SI) that represents the positive influence that influential subjects have over the use of a new technology. Nursing is social, and from the very beginning, that is from the first studies into it, future nurses are exposed to the peer group of nurses that teach a way to do and a way to be [31].
4. Facilitating Conditions (FC) are the reflection of how much an individual believes that his organisation is available to support the use of technology and is actually able to do so. As Nilsen and colleagues say [32], if elements like the opportunity to influence the change, being prepared for it and valuing it are present, the change is more likely to be embraced and then happen.

A first set of hypotheses are, therefore, those related to the base hypotheses of the UTAUT model.

- H1. Performance expectancy about a new technology will have a positive relation with nurses’ intention to adopt it (i.e. nurses’ behavioural intention).
- H2. Effort expectancy about a new technology will have a positive relation with nurses’ intention to adopt it (i.e. nurses’ behavioural intention).
- H3. Social influence will have a positive relation with nurses’ intention to adopt a new technology (i.e. nurses’ behavioural intention).
- H4. Facilitating conditions for the technology implementation will have a positive relation with nurses’ use of the technology (nurses’ use behaviour).
- H5. The intention to use a technology will have a positive relation with nurses’ use of the technology (nurses’ use behaviour).

In the healthcare context, the intensity of such relationships is expected to be influenced by nurses’ age, since the core constructs of the UTAUT model (the determinants of individuals’ behavioural intention to use a technology PE, EE, SI) take on particular characteristics: young nurses do have high performance expectations towards their job as it is particularly hard (both mentally and physically), thus they expect to be rewarded under different points of view. Studies show how recognition is the most important motivator and goal [33], while they seek for stability, professional growth and adequate supervision (Ibidem). The effort expectancy is high, as there’s a wide variety of things to know and abilities to master, but they really can cope is the colleagues help them in a supportive way while providing guidance [34]. Since here a constant always shows: relatedness. The latter is a priority value in nurse professionals, thus SI construct does have a strong influence especially for what novices, young nurses, are concerned.

Each of these relationships are expected to be more intense in young nurses. Consequently, we expect the UTAUT model to have different characteristics for young nurses: we expect the impact of PE, EE, SI to be greater for younger nurses.

Further, recent studies [35] highlight that innovation is a way to motivate and attract staff, especially young people; when a novice starts the journey to become a professional, the close presence and the reassuring support of the seniors is a key factor to retention and motivation [34].

Thus, we expect that nurses’ age will influence the core relationships of the UTAUT model when applied to technology acceptance in technology-intensive healthcare contexts.

- H6. The relationships between use intention, performance expectancy, effort expectancy and social influence will be stronger for younger nurses.
- H7. The impact of facilitating conditions on use behaviour will be stronger for younger nurses.
- H8. The relationship between use intention and use behaviour will be stronger for younger nurses.

## Method

### Data collection

Our empirical setting is based on survey data collected from Italian nurses operating in 6 healthcare departments located in the Tuscany Region, by means of a standardized questionnaire made of twelve questions that operationalize the main constructs of the UTAUT model. The survey was administered through the Qualtrics XM platform in the period August–September 2019. The questionnaire was sent to the healthcare executives of the 6 departments, asking to identify from 5 to 10 nurses assigned to technology-intensive units (i.e. units characterized by the continuous introduction of new technologies, such as robotic surgery units, intensive care units and remote control units for telemetries). The technologies under evaluation were minimally invasive surgery robotics, cardiac remote-control systems, and advanced dressings for complex injuries (including nanotechnology applications and artificial skin devices).

The survey was sent to 62 nurses, and 54 valid responses were considered for the analysis.

The majority of the respondents are women (70%), 44–55 years-old (57%), working mainly in the operating room (60%) and with long seniority (37% with 20–30 years of nursing experience and 28% with more than 30 years).

The web questionnaire includes the scale to measure the UTAUT constructs through multiple items, using a 100-points interval scale (Table 1). The following section details how each UTAUT construct is measured.

### Measures

Performance Expectancy (PE) in high technology contexts is determined by expectations of professional growth, safety and quality of care and efficiency in terms of time savings. Effort Expectancy (EE) is represented by the perceived ease of acquiring the necessary skills to use the high technology and the level of integration of this use in the nurses’ daily practice. Social Influence (SI) reflects the perceived opinion of fellow nurses on how professional the use of high technology is. Facilitating Conditions (FC) are expressed by how great the nurse perceives the support from the entire organization in learning how to use the high technology. As all nurses involved in the study currently work in high technology contexts, Behavioral Intention (BI) and Use Behavior (UB) are reflects how well the nurse has adapted to the technology use and will continue to use high technologies with conviction. The UTAUT-related constructs were estimated through 9 measurement items, by asking respondents to evaluate different statements on a 100-point interval scale (from Totally disagree to Totally agree), see Table 2.

**Table 2:** Measurement items related to the UTAUT constructs

Constructs		Measurement items
Performance expectancy	pe_1_	The use of the new technology is an opportunity to create new paths in my profession
	pe_2_	The use of the new technology is an element that makes me feel more confident about the quality of performance I provide to patients
	pe_3_	Using the new technology enables me to save time
Effort expectancy	ee_1_	I have easily acquired the necessary skills to use the new technology
	ee_2_	The new technology became an integral part of my working life
Social influence	si	My peers think that using the new technology is important to value our profession
Facilitating conditions	fc	My organisation has enabled me to learn how to use new technologies
Behavioural intention	bi	I have adapted with conviction to the use of new technologies
Use behaviour	ub	I will continue to use new technologies with conviction, while continuing to learn

### Data analysis

The three exogenous determinants of BI in the UTAUT model were estimated through a confirmatory MIRT (Multidimensional Item Response Theory) analysis.

In line with the UTAUT theoretical premises, we assume that three distinct latent characteristics underlie nurses’ behavioural intention (BI): Performance Expectancy (PE), Effort Expectancy (EE) and perceived Social Influence (SI) of the introduced technology.

The three determinants of BI were therefore conceived as reflective constructs, which are determined when answers to a set of questions (the observed set of measurement items) *reflect* an underlying latent attitude (i.e. individuals’ performance expectancy, effort expectancy, perceived social influence) rather than *forming* the construct.^1^ As some items might be more relevant than others in discriminating individuals with *different* PE, EE, or perceived SI in different contexts, the use of MIRT allowed investigating in depth the constructs’ factor structure in the context of nurses’ new technology adoption.^2^

**Fig. 1:**
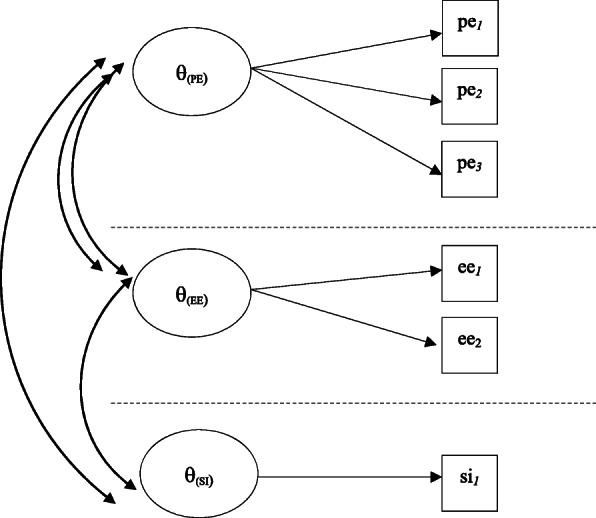
MIRT model specification

In this case, a multiunidimensional latent structure was specified where the three latent characteristics are allowed to correlate [36] (Fig. 1).

For each dimension, a continuous variable representing the individual latent characteristic was estimated through the model (θ_PE_; θ_EE_; θ_SI_). Adding to the individual-level estimates of the latent characteristics, a set of item-level parameters were also estimated to test the external validity of the three constructs.

After the application of MIRT to identify the latent variables for each construct, the UTAUT basic model was tested using the entire dataset.

A two stages regression analysis was first conducted using a Tobit model. In the first stage, the BI equation was tested, including the three exogenous regressors (PE, EE, SI) as well as nurses’ age as a moderator. As it was proposed in the UTAUT model [10] the other two individual characteristics (gender, seniority) were considered as control variables. As a robustness test, the UTAUT model was also validated through a covariance SEM analysis.

The fitted values of BI were used as instruments (endogenous regressors) in the second stage model to estimate the relationship between behavioural intention, facilitating conditions and use behaviour. The coefficients of the UTAUT model were then tested separately for different groups by enforcing constraints for the path coefficients. In a first step, two separate models were run for the different groups without enforcing any constraint (free models) while, in a second step, the models for the two groups were run by setting an equality constraint (i.e. constraining one group by the results of the other). In a third step we tested the group invariance of each path coefficient by comparing the chi2 differences between the groups.

## Results

As results of the MIRT analysis, in Table 3, the values of the discrimination parameters indicate how well each measurement item within the three constructs discriminates among individuals with different levels of PE, EE, SI (i.e. the items’ discriminant validity). Difficulty parameters rather indicate the level of the latent characteristics where each item provides the maximum amount of information (i.e. which items better characterize individuals with high and low levels of PE, EE and SI respectively).

**Table 3:** Confirmatory MIRT analysis results: item-level coefficients

Constructs	Measurement items	Discrimination (α_j_)	Difficulty (β_j_)
Performance expectancy θ_(PE)_	pe_1_	3.353***	4.931***
	pe_2_	6.527***	4.649***
	pe_3_	0.993***	0.353***
Effort expectancy θ(_EE)_	ee1	9.418***	1.020***
	ee_2_	1.443***	1.538***
Social Influence θ(_SI_)	si	11.953***	1.020***

***p < 0.001, **p < 0.01, *p < 0.05

The analysis also allows to theoretically assess the different relevance that the three constructs assume in influencing nurses’ behavioural intention to use the new technology and their effective use, and what factors can be leveraged to enhance this latter.

In the examined context, social influence (SI) shows the highest discriminatory power among the exogenous determinants of behavioural intention (BI) to use the technology. While the perception of having acquired the necessary skills to use the technology has the highest relevance within the effort expectancy construct and acquiring confidence about the quality of performance provided to patients represents the item that discriminates individuals with high and low levels of performance expectancy.

The difficulty parameters confirm that high levels of performance expectancy characterize nurses considering the use of the technology as an opportunity to attain gains in job performance and increases self-confidence.

### An application of the UTAUT model in the context of nurses’ acceptance and use of new technologies

In the first stage of the Tobit regression analysis the BI equation was tested (Table 4).

**Table 4:** Behavioural intention – Tobit regression

	Model 1	Model 2	Model 3	Model 4
PE	15.852***	26.053***	14.914***	16.52***
	*3.858*	*4.983*	*3.982*	*3.610*
EE	11.490***	11.564***	15.795**	12.581***
	*2.892*	*2.694*	*5.699*	*2.727*
SI	0.071	2.682	1.298	11.772*
	*3.95*	*3.784*	*4.171*	*5.490*
Age_class_2	3.257	4.347	4.338	2.018
	*7.934*	*7.419*	*7.988*	*7.422*
Exp_class_2	2.088	−1.404	0.405	4.069
	*7.412*	*7.001*	*7.616*	*6.951*
Gender_1	0.759	0.641	0.517	0.117
	*5.335*	*4.972*	*5.312*	*4.988*
Age_class_2*PE		−16.344**		
		*5.051*		
Age_class_2*EE			−5.411	
			*6.183*	
Age_class_2*SI				−16.49***
				*5.722*
Constant	75.095***	76.604***	75.612***	75.30***
	*5.017*	*4.699*	*5.023*	*4.685*
N	54	54	54	54
Uncensored	53	53	53	53
Left-censored	1	1	1	1
Right-censored	0	0	0	0
LR chi2	53.3***	61.66***	54.07***	61.1***
Pseudo R2	0.11	0.13	0.11	0.13

Standard errors in italics

*** *p* < 0.001, ** *p* < 0.01, *p < 0.05

In Model 1 the main effects were tested, while in Models 2, 3 and 4 the moderating role of age class was tested, using the highest class (above 45 years old) as a baseline. In Model 1 (main effects) we did not observe an effect of SI on the dependent variable (BI), while the influence of PE and EE on BI were high and significant. In Model 2 we clearly observed a positive and consistent moderating effect of age class (β = − 16.34; *p* < 0.001) on the relationship between PE and behavioural intention to use the technology. The negative coefficient observed for the highest age class confirms that this relationship is positive and significant for younger individuals. Model 3 shows no effect of age on the relationship between EE and BI, while Model 4 confirmed a strong and positive effect of age on the relationship between SI and BI.

The fitted values of BI were used as instruments (endogenous regressors) in the second stage model to estimate the relationship between behavioural intention, facilitating conditions and use behaviour, showing that behavioural intention and facilitating conditions positively affected use behaviour (Table 5).

**Table 5:** Use behaviour (UB) – Instrumental variable Tobit regression

	Model 1
BI (instrumented)	0.670***
	*0.132*
FC	0.165*
	*0.095*
Constant	18.95**
	*8.750*
N	54
Left-censored	0
Right-censored	0
Uncensored	54
LRchi2	39.12***
Pseudo R2	0.081

Standard errors in italics

*** *p* < 0.001

To further test our hypotheses and as a robustness test, a covariance SEM analysis was conducted using STATA15. Due to the small sample size robust standard errors were estimated. The values of the overall goodness of fit statistics were satisfactory (chi2 = 149.4 (df = 9), GFI = 0.788 CFI = 0.803, RMSEA 0.361) and all significant at the *p* < 0.001 level.

**Fig. 2:**
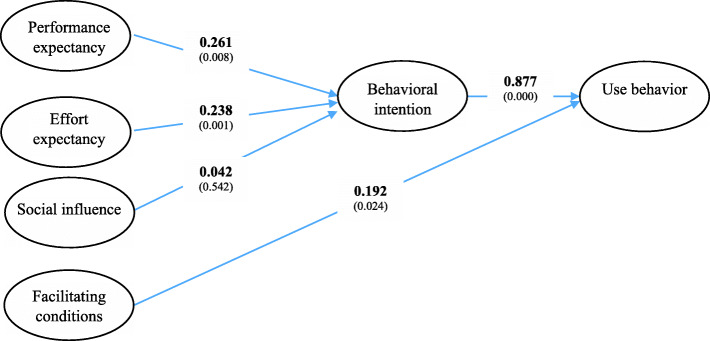
test results of the basic UTAUT model

The test results of the basic UTAUT model for the entire sample showed that all paths were significant, while it confirmed that one path (social influence- > behavioural intention) was not significant (Fig. 2).

The analysis confirmed that performance expectancy and effort expectancy have significant effects on behavioural intention and behavioural intention and facilitating conditions positively affected use behaviour. These results led us to accept H1, H2, H4 and H5 while H3 was not supported.

The coefficients of the UTAUT model were then tested separately for different age groups (Table 6).

**Table 6:** Group comparisons: age classes

	Age class group 1 (< 45)	Age class group 2 (> = 45)
(a) Performance expectancy➔Behavioral Intention	0.374***	0.287**
(b) Effort expectancy➔Behavioral intention	0.436***	0.247**
(c) Social influence➔Behavioral intention	0.412***	−0.062
(d) Facilitating conditions➔Use behavior	0.563***	0.179*
(e) Behavioral intention➔Use behavior	0.858***	0.892***

*** *p* < 0.01, ** *p* < 0.05, * *p* < 0.1

All the paths of the UTAUT model are significant for lower age classes (below 45 years old), while the effect of social influence on behavioural intention, as observed for the full model, was not significant for the higher age class. Further, the magnitude of the path coefficients is significantly higher for the lower age class group, while it was similar for one path (behavioral intention- > Use behavior). These results led us to accept H6, H7 and H8.

## Discussion

The hypotheses test results are summarized in Table 7.

**Table 7:** Hypotheses test results

H1	Performance expectancy about a new technology will have a positive relation with nurses’ intention to adopt it (i.e. nurses’ behavioural intention)	Supported
H2	Effort expectancy about a new technology will have a positive relation with nurses’ intention to adopt it (i.e. nurses’ behavioral intention)	Supported
H3	Social influence will have a positive relation with nurses’ intention to adopt a new technology (i.e. nurses’ behavioral intention)	Not supported
H4	Facilitating conditions for the technology implementation will have a positive relation with nurses’ use of the technology (nurses’ use behaviour)	Supported
H5	The intention to use a technology will have a positive relation with nurses’ use of the technology (nurses’ use behaviour)	Supported
H6	The relationships between use intention, performance expectancy, effort expectancy and social influence will be stronger for younger nurses	Supported
H7	The impact of facilitating conditions on use behaviour will be stronger for younger nurses	Supported
H8	The relationship between use intention and use behaviour will be stronger for younger nurses	Not Supported

Not surprisingly, all the hypotheses concerning the original UTAUT model were confirmed, except for H3. Our results showed a positive and significant effect of performance expectancy and effort expectancy of nurses on their behavioural intention to use a new technology, a positive relationship between nurses’ behavioural intention to use the new technology and nurses’ use behaviour as well as a positive influence of facilitating conditions on nurses’ use behaviour. By contrary, despite social influence represents a relevant component of the exogenous determinants of nurses’ behavioural intention, no significant relationship is found between the two constructs in the full sample. To this point, what is more novel and interesting in our results is the influence of nurses’ age on the model: we find that nurses’ age and experience have a strong impact on the relationships of the UTAUT model applied to technology-intensive healthcare units.

The study pointed out that UTAUT model can be used to explain the acceptance of technology in healthcare technology-intensive contexts, as it is extensively been validated through time and different national contexts [25, 37]. The results of this study point out that, for a particular kind of setting, namely the technology-intensive healthcare units, the age is a moderator for the social influence: it could be argued that social influence, or peer opinion, matters more for young nurses while older nurses seem to do not care about their peer opinion.

A possible explanation lies on the fact that young nurses rely more than the others upon their colleagues while the older nurses feel more confident and are less influenced by the others. For older nurses the expectation of safety and quality of care and efficiency in terms of time savings, as well as the acquiring confidence about the new technology has a stronger relevance in their acceptance of innovations. This in line with the fact the nurses’ ability to manage the technology is gained mainly through experience in the intensive care unit [38] and then the operational aspects of machinery management on patient care are considered as mainly important by nurses. In particular, our analysis of the UTAUT endogenous constructs (Table 6) showed that the perception of having acquired the necessary skills to use the technology has the highest relevance within the effort expectancy construct, as well as acquiring confidence about the quality of performance provided to patients is the most discriminating aspects for the performance expectancy.

The strength of this study is the application of UTAUT model to analyse the acceptance of innovations by nurses in high-tech healthcare contexts, since this theoretical approach has never been applied to this topic. The use of MIRT to identify the latent variables for each construct of UTAUT model helps also to identify which factors should be considered ad leverage to amplify the high technology use by nurses.

A limit of the study is the sample size of nurses involved, but this is also due to limited number of nurses working in the high technology healthcare contexts.

In the specific context of nurses working in high-technology settings UTAUT framework proves itself fit in describing their patterns of acceptance of technologies except as regards one of the four constructs that influence behaviour intention, namely social influence. Peer-relations and peer-dynamics have to be investigated in order to tailor appropriate policies to improve nurses’ compliance in the acceptance of technology. Pandemic served as a catalyst of innovation where fast technology adoption played an important role [39]. Undoubtedly, it posed unique challenges to established working routines. Especially in the peak of the pandemic, nursing staff management required an increase in efficiency (when the pace of work was overwhelming) but at the same time increased readiness in the use of technologies that never were part of prior work. Future empirical research should focus on this emerging context.

Clinical research and technology have been boosted from the easiest ones to the most complex ones. In this light our paper provides a novel empirical example on the dynamics related to the acceptance of nurses for intensive technology. Further investigation may look at the behavioural intention of nurses working in other setting to test if the factors leading nurses to use intensive technology are the same of those already working in such context.

### Practical implications

The healthcare management, at all levels of the organizations, is called to an always greater effectiveness and efficiency, given the shortage of nurses in the world and the complication of the global epidemiological scene. Tools like UTAUT are everything except style exercises: following the information they can provide can be of help in stimulating the intention to use technology, as it can be seen as a factor of attachment and pleasure in carrying out one’s work; therefore, increasing or stimulating the determinants of behavioural intention can be profitable in improving the work environment, hence the satisfaction of nurses. This consideration is true especially since nurses are the professional category that counts the highest percentage of dissatisfied workers greater than satisfied ones [35]. The levers to stimulate these behaviours are different in relation to age. In particular for young people, it is possible to activate personnel’s policies through socialization with the most passionate colleagues (SI) and ad hoc training courses (FC), for others it is possible to leverage on the PE and EE.

## Supplementary Information

**Additional file 1.**

## Data Availability

Data sets and output files of the data analysis are available from the authors. on request.

## Footnotes

1 The implicit assumption characterizing formative constructs (conceived as linear combinations of pre-defined measures) is that the constructs exist only if all the relevant items are present.

2 In models containing multi-item constructs it is important to test the reliability (i.e. the internal validity) of the constructs. The specification of the confirmatory MIRT analysis follows many of the rules in the structural equation modelling framework for Confirmatory Factor Analysis (CFA). However, one important assumption of IRT models implies that item responses are independent (uncorrelated) given a persons’ level of a latent trait; it means that any existing correlation between observed responses to different items are absorbed by the latent construct. Compared to other techniques based on classical test theory (as, for instance, Chronbach alpha’s test), IRT measures show therefore higher reliability as each construct’s internal validity is assessed conditional on the scores of the estimated latent construct. For these reasons, the use of MIRT yields also much reliable parameter estimates in small samples.
