# Supplementary material for: Nurses and the acceptance of innovations in technology-intensive contexts: the need for tailored management strategies
Source: BMC Health Serv Res. 2021 Jul 3;21:639. doi: 10.1186/s12913-021-06628-5 (PMC8253682; doi:10.1186/s12913-021-06628-5)
Supplement: Supplementary file 1 — Additional file 1. [file 12913_2021_6628_MOESM1_ESM.docx]

**Appendix**

Questionnaire used for the analysis:

- How old are you?
- How many years of work experience do you have?
- Can you please indicate your gender?
- In which Hospital do you work?
- In which unit do you work?
- Innovation applied to my work makes my work environment stimulating
  [*from 0 to 100, from totally disagree to totally agree]*
- Innovation applied to my work makes my work environment satisfactory *[from 0 to 100, from totally disagree to totally agree]*
- My peers think that using the new technology is important to value our profession

*[from 0 to 100, from totally disagree to totally agree]*

- The use of the new technology is an opportunity to create new paths in my profession
  *[from 0 to 100, from totally disagree to totally agree]*
- The use of the new technology is an element that makes me feel more confident about the quality of performance I provide to patients
  *[from 0 to 100, from totally disagree to totally agree]*
- Working in an environment with new technologies has necessitated a specific personnel management policy
  *[from 0 to 100, from totally disagree to totally agree]*
- Using the new technology enables me to save time
  *[from 0 to 100, from totally disagree to totally agree]*
- My organisation has enabled me to learn how to use new technologies
  *[from 0 to 100, from totally disagree to totally agree]*
- I have easily acquired the necessary skills to use the new technology
  *[from 0 to 100, from totally disagree to totally agree]*
- The new technology became an integral part of my working life
  *[from 0 to 100, from totally disagree to totally agree]*
- I have adapted with conviction to the use of new technologies
  *[from 0 to 100, from totally disagree to totally agree]*
- I will continue to use new technologies with conviction, while continuing to learn
  *[from 0 to 100, from totally disagree to totally agree]*
